# AI integration in nephrology: evaluating ChatGPT for accurate ICD-10 documentation and coding

**DOI:** 10.3389/frai.2024.1457586

**Published:** 2024-09-02

**Authors:** Yasir Abdelgadir, Charat Thongprayoon, Jing Miao, Supawadee Suppadungsuk, Justin H. Pham, Michael A. Mao, Iasmina M. Craici, Wisit Cheungpasitporn

**Affiliations:** ^1^Division of Nephrology and Hypertension, Mayo Clinic, Rochester, MN, United States; ^2^Chakri Naruebodindra Medical Institute, Faculty of Medicine Ramathibodi Hospital, Mahidol University, Samut Prakan, Thailand; ^3^Mayo Clinic College of Medicine and Science, Mayo Clinic, Rochester, MN, United States; ^4^Division of Nephrology and Hypertension, Department of Medicine, Mayo Clinic, Jacksonville, FL, United States

**Keywords:** AI-assisted coding, ICD-10, nephrology, healthcare reimbursement, clinical workflow efficiency

## Abstract

**Background:**

Accurate ICD-10 coding is crucial for healthcare reimbursement, patient care, and research. AI implementation, like ChatGPT, could improve coding accuracy and reduce physician burden. This study assessed ChatGPT’s performance in identifying ICD-10 codes for nephrology conditions through case scenarios for pre-visit testing.

**Methods:**

Two nephrologists created 100 simulated nephrology cases. ChatGPT versions 3.5 and 4.0 were evaluated by comparing AI-generated ICD-10 codes against predetermined correct codes. Assessments were conducted in two rounds, 2 weeks apart, in April 2024.

**Results:**

In the first round, the accuracy of ChatGPT for assigning correct diagnosis codes was 91 and 99% for version 3.5 and 4.0, respectively. In the second round, the accuracy of ChatGPT for assigning the correct diagnosis code was 87% for version 3.5 and 99% for version 4.0. ChatGPT 4.0 had higher accuracy than ChatGPT 3.5 (*p* = 0.02 and 0.002 for the first and second round respectively). The accuracy did not significantly differ between the two rounds (*p* > 0.05).

**Conclusion:**

ChatGPT 4.0 can significantly improve ICD-10 coding accuracy in nephrology through case scenarios for pre-visit testing, potentially reducing healthcare professionals’ workload. However, the small error percentage underscores the need for ongoing review and improvement of AI systems to ensure accurate reimbursement, optimal patient care, and reliable research data.

## Introduction

The International Classification of Diseases, 10th Revision (ICD-10) coding serves as the standardized language for classifying diseases, injuries, and healthcare procedures, and is published by the World Health Organization (WHO). Based on ICD-10, the Centers for Medicare and Medicaid Services (CMS) and the National Center for Health Statistics (NCHS), two departments within the Department of Health and Human Services (DHHS) provide the guidelines for coding and reporting using the International Classification of Diseases, 10th Revision, Clinical Modification (ICD-10-CM). The ICD-10 coding is essential for reimbursement, healthcare service eligibility, and research (ICD, 2024). Nephrology, the medical specialty focused on kidney diseases, involves intricate conditions that demand precise coding for appropriate treatment and monitoring. Conditions like acute kidney injury (AKI) and chronic kidney disease (CKD) impact individual health and carry substantial healthcare costs.

Accurate ICD-10 coding in nephrology is crucial. It influences reimbursement, the reliability of disease registries, patient care, and the quality of research data. Coding errors can result in improper patient management, billing inaccuracies, and financial losses. Additionally, the complexity of coding nephrology cases can overwhelm physicians, reducing time for patient care and contributing to burnout (Burns et al., 2012; Esteva et al., 2017; Alonso et al., 2020; Lee et al., 2023; Lim et al., 2023; Thirunavukarasu et al., 2023).

The rise of AI and automated clinical coding in medicine offers the potential to improve coding accuracy, efficiency, and cost-effectiveness. Tools like ChatGPT have shown rapid advancement, suggesting they may possess the capabilities to transform how patient data are processed in healthcare (Stanfill et al., 2010; Cook et al., 2019; Stanfill and Marc, 2019; Campbell and Giadresco, 2020; Dong et al., 2022). By handling large, complex datasets with nuance, AI could revolutionize the coding process, alleviating the administrative burden on healthcare professionals while ensuring high-quality, standardized data (Zhong et al., 2023). However, despite AI’s promise, focused studies examining the effectiveness of AI tools in accurately coding complex nephrology cases are limited (Jiang et al., 2017). Most research centers on broader medical conditions or preliminary AI assessments. There is a lack of comparative analyses between AI versions in nephrology real-world clinical coding contexts. This leaves a significant knowledge gap regarding AI’s practical application and reliability in nephrology coding.

This study addresses these gaps by evaluating the performance of two ChatGPT versions (3.5 and 4.0) in ICD-10 coding for nephrology. It assesses the accuracy of AI-generated codes across various simulated nephrology cases, ranging from common conditions like AKI and CKD to more complex diagnoses. The study compares the effectiveness of these AI versions in enhancing coding accuracy, efficiency, and reducing physicians’ administrative workload. By identifying specific nephrology conditions where AI coding may encounter challenges, this research offers direction for further AI refinement and highlights areas where human expertise remains essential (Soroush et al., 2024). Ultimately, this study aims to provide insights into the potential of AI in improving the coding process in nephrology, with implications for better patient care, more accurate reimbursement, and enhanced research quality.

This study offers several key contributions to AI-assisted medical coding in nephrology. It provides the first comprehensive assessment of ChatGPT’s performance in ICD-10 coding for nephrology cases, comparing versions 3.5 and 4.0. Our two-round evaluation offers insights into the consistency of AI-generated codes over time. We identify both the strengths and limitations of AI in coding complex nephrology conditions, guiding future developments. Finally, we discuss the potential impact of AI-assisted coding on nephrology practice, including reduced administrative burden and improved coding accuracy. These insights pave the way for practical implementation of AI in nephrology coding.

## Results

Among 100 simulated clinical cases, ChatGPT 3.5 assigned the correct ICD-10 diagnosis code in 91 (91%) cases, while ChatGPT 4.0 assigned the correct ICD-10 diagnosis code in 99 (99%) in the first round (Table 1).

**Table 1:** The accuracy of ChatGPT 3.5 and 4.0 in assigning ICD-10 diagnosis code.

	ChatGPT 3.5	ChatGPT 4.0	*p*-value^#^
First round	91 (91%)	99 (99%)	0.02
Second round	87 (87%)	99 (99%)	0.002
*p*-value^*^	0.22	1.00	

^#^Between ChatGPT 3.5 and 4.0. ^*^Between first and second round.

**Figure 1:**
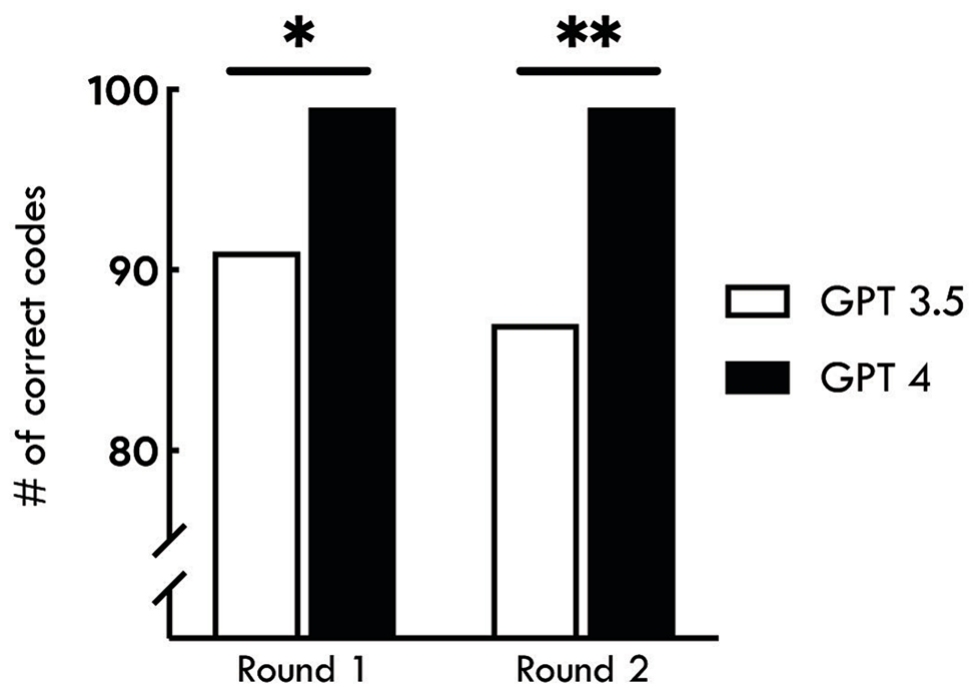
Accuracy of AI-assisted ICD-10 coding for nephrology conditions. The bar graph shows the number of correct ICD-10 codes assigned by ChatGPT versions 3.5 and 4.0 across two rounds of assessments. In Round 1, the accuracy of ChatGPT 3.5 was 91%, while ChatGPT 4.0 achieved 99% accuracy (^*^p = 0.02). In Round 2, both versions showed a slight decrease, with ChatGPT 3.5 at 87% and ChatGPT 4.0 maintaining 99% accuracy (^**^p = 0.002). The results indicate a significantly higher performance of ChatGPT 4.0 compared to ChatGPT 3.5 in both rounds. The accuracy did not significantly differ between the two rounds (*p* > 0.05).

In the second round, ChatGPT 3.5 assigned the correct ICD-10 diagnosis code in 87 (87%) cases, while ChatGPT 4.0 assigned the correct ICD-10 diagnosis code in 99 (99%) cases. The accuracy of ChatGPT 4.0 was higher than ChatGPT 3.5 (*p* = 0.02 in the first round, and 0.002 in the second round). There was no significant difference in accuracy within ChatGPT version between the first and second rounds (*p* = 0.22 for ChatGPT 3.5 and 1.00 for ChatGPT 4.0) (Figure 1).

Chat GPT 3.5. performance did not do very well with GN diagnoses ICD 10 code, for example, it did not get FSGS diagnosis in both attempts, the same was true for obstructive uropathy due to benign prostatic hyperplasia (BPH) and Acute Emphysematous Pyelonephritis. Also, it struggled with some diagnoses of the ICD-10 code in the first test, like acute pyelonephritis with abscess, but got the right ICD-10 code in the second test in 2 weeks. Interestingly, it got some ICD 10 codes right in the first test, for example, calculus of the ureter, but got it wrong in the second test. In comparison, Chat GPT 4 performance was not just significantly superior but also consistent. It had the same mistake in the two tests, which is Bartter syndrome ICD 10 code.

## Discussion

Recently, Soroush et al. (2024) evaluated the performance of large language models (LLMs) including GPT3.5, GPT-4, Gemini Pro, and Llama2-70b Chat in generating medical billing codes (ICD-9-CM, ICD-10-CM, and CPT) when given code descriptions. All tested LLMs underperformed, with GPT-4, the top performer, achieving less than 50% exact match rates. GPT-4 led with exact match rates of 45.9% (ICD9-CM), 33.9% (ICD-10-CM), and 49.8% (CPT), while Llama2-70b Chat trailed with rates below 3% across all systems. The study revealed that LLMs often generated codes that were conceptually similar but lacked the precision required for clinical use, sometimes producing generalized or even fabricated codes. Higher accuracy correlated with frequently used codes, shorter code lengths, and concise descriptions. LLMs demonstrated superior performance on ICD-9-CM codes compared to the more intricate ICD-10-CM codes. The study concluded that base LLMs are inadequate for medical code mapping, emphasizing the need for research into handling newer, more complex ICD structures to enhance LLM performance in this domain (Soroush et al., 2024).

However, the findings of our study demonstrate a significant advancement in AI-assisted ICD-10 coding for nephrology, with results that contrast sharply with those of broader medical coding studies. Our research, which focused specifically on nephrology-related ICD-10 codes, showed that ChatGPT 4.0 achieved a consistent 99% accuracy across two evaluation rounds, while ChatGPT 3.5 performed at 91 and 87% accuracy in the first and second rounds, respectively. These high accuracy rates differ markedly from the lower performance reported in more general studies, such as Soroush et al.’s work. Several key factors (Table 2) likely contribute to our improved outcomes: (1) Our study’s focus on nephrology specific ICD-10 coding, as opposed to the broader scope across multiple coding systems in Soroush et al.’s work. (2) Our use of clinical scenarios mimicking pre-visit testing information, which aligns more closely with real-world practice, versus the official code descriptions used by Soroush et al. (2024). (3) Our requirement for a single, most appropriate ICD-10 code, allowing for more interpretative flexibility than the exact code matching in Soroush et al.’s study. (4) Our use of a simple, clinically relevant prompt design. (5) Potential improvements in the most recent versions of ChatGPT, particularly GPT-4, used in our study. While we observed some persistent challenges with complex nephrology conditions, our results suggest that AI, particularly advanced models like ChatGPT 4.0, has significant potential to reduce administrative burden in specialty-specific medical coding while maintaining high accuracy. These findings underscore the importance of tailoring AI applications to specific medical specialties and clinical contexts for optimal performance.

**Table 2:** Comparison of AI-assisted medical coding studies.

Aspect	Study of Soroush et al. (2024)	Our study
Focus	Broad medical coding (ICD-9-CM, ICD-10-CM, and CPT)	Nephrology-specific ICD-10 coding
AI models	GPT-3.5, GPT-4, Gemini Pro, and Llama2-70b Chat	ChatGPT 3.5 and 4.0
Input format	Official code descriptions	Clinical scenarios mimicking pre-visit testing
Task	Generate exact matching codes	Identify single most appropriate ICD-10 code
Prompt design	Standardized for code generation	Simple, clinically relevant
Top performance	GPT-4: 45.9% (ICD-9-CM), 33.9% (ICD-10-CM), and 49.8% (CPT)	ChatGPT 4.0: 99% (ICD-10 for nephrology)
Performance range	Below 3% to below 50%	87–99%
Code types	Multiple (ICD-9-CM, ICD-10-CM, and CPT)	Single (ICD-10)
Specialty focus	General medical	Nephrology-specific
Main finding	Base LLMs inadequate for medical coding	AI shows high potential for specialty-specific coding.
Accuracy factors	Code frequency, length, description conciseness	Specialty focus, clinical context, latest AI versions.
Conclusion	Need for further research on complex ICD structures	AI can reduce administrative burden in specialty coding through Nephrology case scenarios for pre-visit testing.

Despite the impressive overall improvement, both ChatGPT versions demonstrated repeated inaccuracies with specific complex nephrology conditions (Liopyris et al., 2022). Version 3.5 struggled with conditions like Obstructive Uropathy due to Benign Prostatic Hyperplasia and Acute Emphysematous Pyelonephritis, while version 4.0 had consistent difficulty with Bartter Syndrome. These conditions involve intricate pathophysiology and require careful consideration of multiple factors for accurate diagnosis and coding. The challenges faced by AI in these cases underscore the complexity of nephrology and the need for AI systems to grasp the subtle nuances that distinguish these conditions. While AI-assisted coding is promising, it still requires refinement, particularly for less common or complex renal diseases. The superior performance of ChatGPT 4.0 is likely due to its enhanced algorithms, larger training datasets, and improved comprehension of nephrology-specific terminology. However, the persistent errors highlight the importance of specialized AI training on the intricacies of rare kidney disorders and their associated complications.

The cost-effectiveness of AI-assisted coding is a crucial consideration in nephrology. Accurate coding is essential for proper reimbursement, given the high costs associated with renal replacement therapies, transplantation, and the management of CKD complications. Improved coding accuracy and reduced administrative burden on nephrologists could lead to significant cost savings for healthcare institutions. However, the successful implementation of AI tools requires proper training for nephrologists and other renal healthcare professionals. This includes not only technical training on using AI-assisted coding systems but also education on the limitations and potential biases of AI in the context of kidney disease diagnosis and management.

Ethical considerations surrounding AI in nephrology must also be addressed. While our study used simulated data to protect patient privacy, the potential for algorithmic bias and the importance of transparency in AI decision-making processes should be explored further in the context of kidney disease. Nephrology involves complex decision-making, often in the face of comorbidities and socioeconomic determinants of health. Ensuring that AI tools do not perpetuate or exacerbate existing health disparities in kidney care is crucial. Moreover, the development and refinement of AI tools for nephrology coding require close collaboration between nephrologists, data scientists, and AI experts. Fostering such interdisciplinary partnerships is vital to ensure the creation of clinically relevant and reliable AI systems that account for the unique challenges in renal care. As AI becomes more integrated into nephrology workflows, regulatory bodies will play a vital role in ensuring their safety, efficacy, and ethical use, necessitating clear guidelines and oversight mechanisms specific to the use of AI in kidney disease management (Abdullah et al., 2021; Rajpurkar et al., 2022; Gordon et al., 2024).

It is important to acknowledge that our study’s reliance on simulated cases may not fully mirror the complexity of real-world patient encounters. Additionally, we focused on a specific AI toolset without broader comparisons to other technologies or traditional coding methods. These factors could limit the generalizability of our findings. Nonetheless, the results are encouraging and suggest significant potential for AI-assisted coding. Further research should prioritize expanding AI training datasets to encompass a wider range of nephrology cases, particularly those that are rare or complex. Additionally, real-world clinical trials would provide more robust evidence for AI’s practical benefits. To optimize outcomes, a hybrid approach combining AI with human oversight is likely the best path forward (Chen et al., 2019). This would leverage AI’s speed and efficiency while ensuring the nuanced understanding of medical experts. Such a system could advance patient care by ensuring precise documentation, which is essential for accurate treatment planning. Our use of simulated data was an ethical choice that bypassed concerns around patient privacy, allowing us to explore new technological applications in healthcare without compromising patient confidentiality.

Our study specifically focused on initial coding at the time of nephrology referral, using brief case scenarios to improve workflow efficiency and reduce administrative burden. We acknowledge that this approach, focusing on a single correct answer, simplifies real-world coding practices. While this aligns with our goal of streamlining initial referrals, we recognize that comprehensive nephrology care often requires multiple ICD-10 codes to accurately represent a patient’s full clinical picture. Future studies are needed to explore AI applications in more complex coding scenarios, such as generating multiple codes for detailed clinical presentations at later care stages, handling comorbidities, and adapting to evolving patient conditions. These future research directions could involve developing AI models capable of suggesting primary and secondary diagnosis codes, thus addressing the full complexity of nephrology coding practices.

The findings of our study revealed that the performance reduction was primarily observed in ChatGPT 3.5, while ChatGPT 4.0 maintained consistent performance across both rounds. This variation could be attributed to the inherent stochasticity of LLMs, potential differences in case complexity challenging for version 3.5, and the lack of controlled parameters in our initial design. To address these issues in future studies, especially for ChatGPT 3.5, we propose several methodological improvements: utilizing the OpenAI API for precise parameter control, setting consistent temperature, implementing role assignment through system prompts, using fixed random seeds for reproducibility, and conducting multiple runs to report average performance (Miao et al., 2024b).

We acknowledge the potential challenges of out-of-vocabulary issues and incorporating newly added ICD-10 codes in GPT models (Kaur et al., 2023). Our study focused on well-established ICD-10 codes used during the training periods of GPT 3.5 and 4.0. For future implementations addressing newer updates, we propose several strategies: (1) Regular model fine-tuning with the latest ICD-10 updates, (2) A hybrid approach combining AI outputs with an updated ICD-10 database for cross-checking, and (3) Retrieval augmented generation, allowing real-time access to the most current ICD-10 information (Miao et al., 2024a,b). These approaches could mitigate issues with out-of-vocabulary or newly added codes, ensuring the AI system remains current and clinically relevant. Future studies should explore these approaches, particularly focusing on retrieval augmented generation, to enhance the model’s ability to handle the most recent ICD-10 updates. This could involve integrating an external, regularly updated ICD-10 database that the model can query during the code generation process.

**Figure 2:**
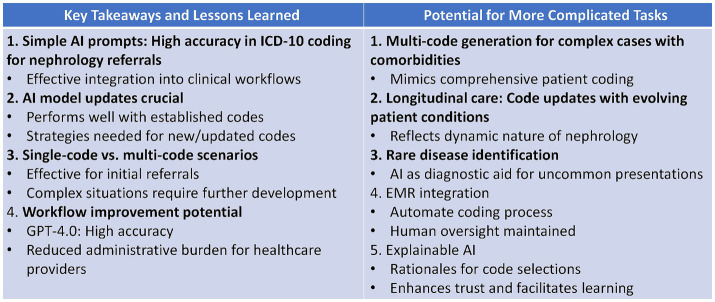
Key takeaways and potential future directions for AI-assisted ICD-10 coding in nephrology.

Our study demonstrated AI’s potential for accurate ICD-10 coding in nephrology referrals using simple prompts, suggesting easy clinical integration (Figure 2). While effective for initial single-code scenarios, we recognize the need for strategies to handle new codes and more complex, multi-code situations. Future research could explore AI’s capability for multi-code generation, longitudinal care coding, rare disease identification, EMR integration, and explainable AI. These advancements could further reduce administrative burden, improve comprehensive patient coding, and enhance trust in AI-assisted medical coding.

In conclusion, our study provides compelling evidence that advanced AI tools like ChatGPT 4.0 offer substantial improvements in coding accuracy, potentially revolutionizing clinical workflows in nephrology. While our findings focused on nephrology, the potential for AI-assisted coding likely extends to other medical specialties with complex terminology and coding requirements. While specific coding challenges remain, continued refinement of AI algorithms and training datasets holds immense promise. As AI evolves, it has the potential to become indispensable in medicine, supporting more efficient, accurate, and patient-centered healthcare. The significant benefits of AI-assisted ICD-10 coding in nephrology point toward a future of healthcare where technology enhances reimbursement, care delivery, research, and physician well-being.

## Methods

### Study design

The research was structured as an experiment involving simulated clinical cases. It compared the coding outputs from ChatGPT against a standard set of correct codes predetermined by expert nephrologists.

### Development of simulated cases

Two board-certified nephrologists (CT and WC) with over 5 years of clinical experience collaboratively developed 100 simulated patient cases in Nephrology settings incorporating case scenarios and pre-visit testing data. These cases were developed to cover a range of common conditions seen in nephrology settings. The two nephrologists discussed and assigned the most suitable ICD-10 diagnosis code for each simulated case.

### Evaluation procedure

ChatGPT 3.5 and 4.0 were independently asked to assign an ICD-10 diagnosis code to each of the 100 simulated clinical cases. Each case scenario was entered into ChatGPT in the narrative form, and was queried “What is the most suitable ICD-10 diagnosis code for this case” (White et al., 2023).

Rationale for Simple Prompting: Our primary goal in this study was to evaluate the performance of ChatGPT in a setting that closely mimics real-world clinical practice. We specifically chose a simple, straightforward prompt for several reasons:

1. Clinical practicality: We aimed to assess how ChatGPT would perform with the type of quick, concise queries that healthcare professionals are likely to use in their daily workflow.
2. Reducing cognitive load: One of our key objectives was to explore ways to “reduce the burden on physicians, who often struggle with the complexity and time-consuming nature of coding tasks.” A simple prompt aligns with this goal by minimizing the time and effort required from healthcare staff to interact with the AI system.
3. Accessibility: We wanted to evaluate a method that could be easily adopted by various healthcare staff members, not just those with extensive training in AI interactions.
4. Workflow integration: Our approach was designed to seamlessly integrate into existing clinical workflows without adding additional steps or complexity.

The ICD-10 diagnosis code generated by ChatGPT was compared against the correct diagnosis codes predetermined by the nephrologists who designed the cases. The nephrologists were masked from ChatGPT’s response and vice versa. The evaluation of ChatGPT’s performance in the same set of 100 simulated cases was conducted in two separate rounds with a 2-week interval in April 2024 to observe the reliability of ChatGPT’s performance over time.

### Statistical analysis

The accuracy of ChatGPT in assigning the correct ICD-10 diagnosis code was reported. The difference in accuracy between ChatGPT 3.5 and 4.0 and between the first round and second round were tested using McNemar’s test. The statistical analyses was performed using the JMP statistical software (version 17.0, Cary, NC).

## Data Availability

The original contributions presented in the study are included in the article/Supplementary material; further inquiries can be directed to the corresponding author.

## References

[ref1] Abdullah Y. I. Schuman J. S. Shabsigh R. Caplan A. Al-Aswad L. A. (2021). Ethics of artificial intelligence in medicine and ophthalmology. Asia Pac J Ophthalmol (Phila) 10, 289–298. doi: 10.1097/apo.0000000000000397, PMID: 34383720 PMC9167644

[ref2] Alonso V. Santos J. V. Pinto M. Ferreira J. Lema I. Lopes F.. (2020). Problems and barriers during the process of clinical coding: a focus group study of coders' perceptions. J. Med. Syst. 44:62. doi: 10.1007/s10916-020-1532-x, PMID: 32036459

[ref3] Burns E. M. Rigby E. Mamidanna R. Bottle A. Aylin P. Ziprin P.. (2012). Systematic review of discharge coding accuracy. J. Public Health (Oxf.) 34, 138–148. doi: 10.1093/pubmed/fdr054, PMID: 21795302 PMC3285117

[ref4] Campbell S. Giadresco K. (2020). Computer-assisted clinical coding: a narrative review of the literature on its benefits, limitations, implementation and impact on clinical coding professionals. Health Inf. Manag. 49, 5–18. doi: 10.1177/1833358319851305, PMID: 31159578

[ref5] Chen I. Y. Szolovits P. Ghassemi M. (2019). Can AI help reduce disparities in general medical and mental health care? AMA J. Ethics 21, E167–E179. doi: 10.1001/amajethics.2019.167 30794127

[ref6] Cook M. J. Yao L. Wang X. (2019). Facilitating accurate health provider directories using natural language processing. BMC Med. Inform. Decis. Mak. 19:80. doi: 10.1186/s12911-019-0788-x, PMID: 30943977 PMC6448184

[ref7] Dong H. Falis M. Whiteley W. Alex B. Matterson J. Ji S.. (2022). Automated clinical coding: what, why, and where we are? NPJ digital medicine 5, 1–8. doi: 10.1038/s41746-022-00705-7, PMID: 36273236 PMC9588058

[ref8] Esteva A. Kuprel B. Novoa R. A. Ko J. Swetter S. M. Blau H. M.. (2017). Dermatologist-level classification of skin cancer with deep neural networks. Nature 542, 115–118. doi: 10.1038/nature21056, PMID: 28117445 PMC8382232

[ref9] Gordon E. R. Trager M. H. Kontos D. Weng C. Geskin L. J. Dugdale L. S.. (2024). Ethical considerations for artificial intelligence in dermatology: a scoping review. Br. J. Dermatol. 190, 789–797. doi: 10.1093/bjd/ljae040, PMID: 38330217

[ref10] ICD (2024). ICD-10-CM—International Classification of Diseases, (ICD-10-CM/PCS Transition.

[ref11] Jiang F. Jiang Y. Zhi H. Dong Y. Li H. Ma S.. (2017). Artificial intelligence in healthcare: past, present and future. Stroke Vasc Neurol 2, 230–243. doi: 10.1136/svn-2017-000101, PMID: 29507784 PMC5829945

[ref12] Kaur R. Ginige J. A. Obst O. (2023). AI-based ICD coding and classification approaches using discharge summaries: a systematic literature review. Expert Syst. Appl. 213:118997. doi: 10.1016/j.eswa.2022.118997

[ref13] Lee P. Bubeck S. Petro J. (2023). Benefits, limits, and risks of GPT-4 as an AI Chatbot for medicine. N. Engl. J. Med. 388, 1233–1239. doi: 10.1056/NEJMsr2214184, PMID: 36988602

[ref14] Lim Z. W. Pushpanathan K. Yew S. M. E. Lai Y. Sun C. H. Lam J. S. H.. (2023). Benchmarking large language models’ performances for myopia care: a comparative analysis of ChatGPT-3.5, ChatGPT-4.0, and Google bard. EBioMedicine 95:104770. doi: 10.1016/j.ebiom.2023.104770, PMID: 37625267 PMC10470220

[ref15] Liopyris K. Gregoriou S. Dias J. Stratigos A. J. (2022). Artificial intelligence in dermatology: challenges and perspectives. Dermatol Ther (Heidelb) 12, 2637–2651. doi: 10.1007/s13555-022-00833-8, PMID: 36306100 PMC9674813

[ref16] Miao J. Thongprayoon C. Craici I. M. Cheungpasitporn W. (2024a). How to improve ChatGPT performance for nephrologists: a technique guide. J. Nephrol. doi: 10.1007/s40620-024-01974-z, PMID: 38771519

[ref17] Miao J. Thongprayoon C. Suppadungsuk S. Garcia Valencia O. A. Cheungpasitporn W. (2024b). Integrating retrieval-augmented generation with large language models in nephrology: advancing practical applications. Medicina (Kaunas) 60:445. doi: 10.3390/medicina60030445, PMID: 38541171 PMC10972059

[ref18] Rajpurkar P. Chen E. Banerjee O. Topol E. J. (2022). AI in health and medicine. Nat. Med. 28, 31–38. doi: 10.1038/s41591-021-01614-0 35058619

[ref19] Soroush A. Glicksberg B. S. Zimlichman E. Barash Y. Freeman R. Charney A. W.. (2024). Large language models are poor medical coders—benchmarking of medical code querying. NEJM AI 1. doi: 10.1056/AIdbp2300040

[ref20] Stanfill M. H. Marc D. T. (2019). Health information management: implications of artificial intelligence on healthcare data and information management. Yearb. Med. Inform. 28, 056–064. doi: 10.1055/s-0039-1677913, PMID: 31419816 PMC6697524

[ref21] Stanfill M. H. Williams M. Fenton S. H. Jenders R. A. Hersh W. R. (2010). A systematic literature review of automated clinical coding and classification systems. J. Am. Med. Inform. Assoc. 17, 646–651. doi: 10.1136/jamia.2009.001024, PMID: 20962126 PMC3000748

[ref22] Thirunavukarasu A. J. Ting D. S. J. Elangovan K. Gutierrez L. Tan T. F. Ting D. S. W. (2023). Large language models in medicine. Nat. Med. 29, 1930–1940. doi: 10.1038/s41591-023-02448-8, PMID: 37460753

[ref23] White J. Fu Q Hays S Sandborn M Olea C Gilbert H. (2023). A prompt pattern catalog to enhance prompt engineering with ChatGPT. arXiv [Preprint]. doi: 10.48550/arXiv.2302.11382

[ref24] Zhong Q. Ding L. Liu J. Du B. Tao D. (2023). Can ChatGPT understand too? A comparative study on ChatGPT and fine-tuned BERT. arXiv [Preprint]. doi: 10.48550/arXiv.2302.10198

