# Supplementary material for: AI integration in nephrology: evaluating ChatGPT for accurate ICD-10 documentation and coding
Source: Front Artif Intell. 2024 Sep 2;7:1457586. doi: 10.3389/frai.2024.1457586 (PMC11402808; doi:10.3389/frai.2024.1457586)
Supplement: Supplementary file 1 [file Data_Sheet_1.pdf]

Online Supplementary

1. A 45-year-old male presents with severe flank pain and hematuria. CT scan reveals multiple cysts in both kidneys. Diagnosed with Polycystic Kidney Disease.

2. A 62-year-old male with a history of diabetes presents with nausea, vomiting, and decreased urine output. Lab results indicate acute kidney injury. Diagnosed with Acute Kidney Failure.

3. A 35-year-old female presents with joint pain, fever, and a butterfly-shaped rash on her face.(add had rising creatinine and hematuria) Biopsy confirms Lupus Nephritis.

4. A 55-year-old male with hypertension complains of swelling in his legs and fatigue. Urine tests reveal proteinuria. Low serum albumin) Diagnosed with Nephrotic Syndrome with unspecified morphologic changes.

5. A 10-year-old boy presents with failure to thrive, polyuria, and polydipsia. Genetic testing confirms a diagnosis of Cystinosis.

6. A 42-year-old female with a family history of kidney disease, who was diagnosed with hypertension, presented with complaints of abdominal pain and blood in the urine. An ultrasound (US) revealed multiple cysts. She has been diagnosed with Autosomal Dominant Polycystic Kidney Disease (ADPKD).

7. A 25-year-old male with a family history of kidney disease presents with hemolytic anemia, thrombocytopenia, and acute kidney failure. He has been diagnosed with atypical Hemolytic Uremic Syndrome (aHUS).

8. A 8-year-old girl presents with dark urine and joint pain. Genetic testing confirms Alkaptonuria.

9. A 20-year-old male presents with hematuria, sensorineural hearing loss, and eye abnormalities. Diagnosed with X-linked Alport Syndrome.

10. A 65-year-old female with a history of hypertension and diabetes complains of fatigue and nausea. Lab results show elevated creatinine levels showing eGFR of 18. Diagnosed with Chronic Kidney Disease, stage 4.

11. A 30-year-old female presents with muscle weakness, spasms, and fatigue. Blood tests reveal hypokalemia and metabolic alkalosis. Diagnosed with Bartter Syndrome.

12. A 40-year-old male presents with abdominal pain, nausea, and vomiting. CT scan reveals a large renal stone. Diagnosed with Nephrolithiasis.

13. An 18-year-old female presents with edema, oliguria, proteinuria, hematuria, joint pain, skin rash, and peripheral neuropathy. A renal biopsy reveals diffuse proliferative glomerulonephritis. She has been diagnosed with diffuse Lupus Nephritis.

14. A 35-year-old female presents with flank pain, fever, and dysuria. Urine culture shows E. coli growth. Diagnosed with Acute Pyelonephritis.
15. A 60-year-old male with a history of diabetes complains of numbness in his feet and decreased urine output. Diagnosed with Diabetic Nephropathy.
16. A 28-year-old female presents with edema, proteinuria, and microscopic hematuria. Renal biopsy shows focal segmental glomerulosclerosis. Diagnosed with Nephrotic Syndrome with focal and segmental glomerular lesions.
17. A 55-year-old female complains of fatigue, muscle cramps, and itchy skin. Blood tests show elevated creatinine and urea levels. Diagnosed with End-Stage Renal Disease.
18. A 30-year-old male presents with flank pain, hematuria, and a palpable abdominal mass. A CT scan reveals a large renal mass. He has been diagnosed with Malignant Neoplasm of the Kidney, except for the renal pelvis.
19. A 42-year-old female with a history of lupus presents with joint pain, fever, and decreased urine output. Renal biopsy shows diffuse proliferative glomerulonephritis. Diagnosed with Lupus Nephritis.
20. A 60-year-old male presents with flank pain, hematuria, and a history of smoking. CT scan reveals a mass in the renal pelvis. Diagnosed with Malignant Neoplasm of Renal Pelvis.
21. A 65-year-old male with a history of prostate cancer complains of back pain and decreased urine output. CT scan reveals bilateral hydronephrosis. Diagnosed with Obstructive Uropathy.
22. A 30-year-old male with a history of nephrolithiasis presents with severe flank pain and vomiting. CT scan reveals a 5mm obstructing ureteral stone. Diagnosed with Calculus of Ureter.
23. A 45-year-old female presents with hematuria, flank pain, and a palpable abdominal mass. CT scan reveals a large renal oncocytoma. Diagnosed with Benign Neoplasm of Kidney.
24. A 60-year-old male with a history of diabetes and hypertension complains of nocturia, hesitancy, and weak urinary stream. Diagnosed with Benign Prostatic Hyperplasia with Lower Urinary Tract Symptoms.
25. A 28-year-old female presents with dysuria, frequency, and suprapubic pain. Urine culture reveals E. coli. Diagnosed with Acute Cystitis.
26. A 35-year-old female complains of dysuria, frequency, and hematuria. Cystoscopy reveals a small, papillary tumor in the bladder. Diagnosed with Papillary Urothelial Carcinoma of Bladder.
27. A 65-year-old male with a history of smoking complains of gross hematuria and weight loss. Cystoscopy reveals a large, irregular bladder tumor. Diagnosed with Muscle Invasive Bladder Cancer.

28. A 35-year-old male presents with gross hematuria and left flank pain. CT urography reveals a filling defect in the left renal pelvis. Diagnosed with Transitional Cell Carcinoma of Renal Pelvis.
29. A 45-year-old male presents with left flank pain and hematuria. CT scan reveals a 1cm renal mass. Biopsy shows oncocytoma. Diagnosed with Benign Neoplasm of Kidney.
30. A 58-year-old male with a history of hypertension complains of nocturia, hesitancy, and weak urinary stream. Digital rectal exam reveals an enlarged prostate. Diagnosed with Benign Prostatic Hyperplasia.
31. A 60-year-old female with a history of diabetes complains of frequency, urgency, and suprapubic pain. Urine culture reveals Candida albicans. Diagnosed with Candidal Cystitis.
32. A 55-year-old male with a history of hypertension presents with nocturia, hesitancy, and weak urinary stream. Ultrasound reveals bilateral hydronephrosis. Diagnosed with Obstructive Uropathy due to Benign Prostatic Hyperplasia.
33. A 30-year-old female presents with dysuria, frequency, and hematuria. Cystoscopy reveals a small, sessile tumor in the bladder. Biopsy shows low-grade papillary urothelial carcinoma. Diagnosed with Non-Muscle Invasive Bladder Cancer.
34. A 50-year-old male with a history of gout presents with severe left flank pain and hematuria. CT scan shows a 3mm obstructing uric acid stone in the left ureter. Diagnosed with Calculus of Ureter.
35. A 62-year-old female with a history of recurrent urinary tract infections complains of fever, chills, and right flank pain. CT scan reveals a 2cm renal abscess. Diagnosed with Acute Pyelonephritis with abscess.
36. A 35-year-old male presents with nocturia, hesitancy, and weak urinary stream. Digital rectal exam reveals an enlarged prostate. Diagnosed with Benign Prostatic Hyperplasia.
37. A 28-year-old female with a history of diabetes presents with dysuria, frequency, and suprapubic pain. Urine culture reveals Candida albicans. Diagnosed with Candidal Cystitis.
38. A 55-year-old male with a history of nephrolithiasis presents with severe right flank pain radiating to the groin. CT scan shows a 4mm obstructing calcium oxalate stone in the right ureter. Diagnosed with Calculus of Ureter.
39. A 48-year-old female presents with gross hematuria and left flank pain. Cystoscopy reveals a large, sessile tumor in the bladder. Biopsy shows high-grade papillary urothelial carcinoma. Diagnosed with Muscle Invasive Bladder Cancer.
40. A 60-year-old male with a history of smoking presents with gross hematuria and weight loss. CT urography reveals a filling defect in the right renal pelvis. Diagnosed with Transitional Cell Carcinoma of Renal Pelvis.

41. A 30-year-old female presents with dysuria, frequency, and hematuria. Urine culture reveals Escherichia coli. Diagnosed with Acute Cystitis.

42. A 42-year-old male with a history of gout presents with severe left flank pain and vomiting. CT scan shows a 5mm obstructing uric acid stone in the left kidney. Diagnosed with Calculus of Kidney.

43. A 58-year-old female with a history of diabetes complains of right flank pain and fever. CT scan reveals emphysematous pyelonephritis. Diagnosed with Acute Emphysematous Pyelonephritis.

44. A 45-year-old male with a history of recurrent urinary tract infections presents with fever, chills, and left flank pain. Urine culture shows Pseudomonas aeruginosa. Diagnosed with Acute Pyelonephritis.

45. A 35-year-old female presents with severe right flank pain radiating to the groin. CT scan reveals a 3mm obstructing calcium oxalate stone in the right ureter. Diagnosed with Calculus of Ureter.

46. A 62-year-old male with a history of hypertension complains of nocturia, hesitancy, and weak urinary stream. Ultrasound reveals bilateral hydronephrosis. Diagnosed with Obstructive Uropathy due to Benign Prostatic Hyperplasia.

47. A 28-year-old female presents with dysuria, frequency, and suprapubic pain. Urine culture reveals Staphylococcus saprophyticus. Diagnosed with Acute Cystitis.

48. A 50-year-old male with a history of smoking presents with gross hematuria and flank pain. Cystoscopy reveals a small, papillary tumor in the bladder. Biopsy shows low-grade papillary urothelial carcinoma. Diagnosed with Non-Muscle Invasive Bladder Cancer.

49. A 55-year-old female with a history of nephrolithiasis presents with severe left flank pain and vomiting. CT scan shows a 6mm obstructing struvite stone in the left kidney. Diagnosed with Calculus of Kidney.

50. A 55-year-old female with a history of recurrent urinary tract infections presents with fever, chills, and left flank pain. Urine culture shows Pseudomonas aeruginosa. Diagnosed with Acute Pyelonephritis.

51. A 30-year-old male presents with severe right flank pain radiating to the groin. CT scan reveals a 3mm obstructing calcium oxalate stone in the right ureter. Diagnosed with Calculus of Ureter.

52. A 62-year-old male with a history of smoking presents with gross hematuria and flank pain. Cystoscopy reveals a large, sessile tumor in the bladder. Biopsy shows high-grade papillary urothelial carcinoma. Diagnosed with Muscle Invasive Bladder Cancer.

53. A 48-year-old female with a history of nephrolithiasis presents with severe left flank pain and vomiting. CT scan shows a 5mm obstructing struvite stone in the left kidney. Diagnosed with Calculus of Kidney.

54. A 35-year-old male presents with dysuria, frequency, and suprapubic pain. Urine culture reveals Staphylococcus saprophyticus. Diagnosed with Acute Cystitis.
55. A 60-year-old female with a history of diabetes complains of right flank pain and fever. CT scan reveals emphysematous pyelonephritis. Diagnosed with Acute Emphysematous Pyelonephritis.
56. A 28-year-old female with a history of recurrent urinary tract infections presents with fever, chills, and right flank pain. Urine culture shows Klebsiella pneumoniae. Diagnosed with Acute Pyelonephritis.
57. A 50-year-old male presents with severe left flank pain and hematuria. CT scan reveals a 4mm obstructing uric acid stone in the left ureter. Diagnosed with Calculus of Ureter.
58. A 55-year-old male with a history of hypertension complains of nocturia, hesitancy, and weak urinary stream. Ultrasound reveals bilateral hydronephrosis. Diagnosed with Obstructive Uropathy due to Benign Prostatic Hyperplasia.
59. A 42-year-old female presents with dysuria, frequency, and hematuria. Cystoscopy reveals a small, papillary tumor in the bladder. Biopsy shows low-grade papillary urothelial carcinoma. Diagnosed with Non-Muscle Invasive Bladder Cancer.
60. A 65-year-old male with a history of diabetes presents with fever, chills, and left flank pain. Urine culture shows Escherichia coli. Diagnosed with Acute Pyelonephritis.
61. A 30-year-old female with a history of nephrolithiasis presents with severe right flank pain and vomiting. CT scan reveals a 6mm obstructing calcium phosphate stone in the right kidney. Diagnosed with Calculus of Kidney.
62. A 58-year-old male with a history of smoking presents with gross hematuria and weight loss. CT urography reveals a large, irregular tumor in the left renal pelvis. Diagnosed with Transitional Cell Carcinoma of Renal Pelvis.
63. A 45-year-old female presents with dysuria, frequency, and suprapubic pain. Urine culture reveals Proteus mirabilis. Diagnosed with Acute Cystitis.
64. A 50-year-old male with a history of gout presents with severe left flank pain and hematuria. CT scan shows a 3mm obstructing uric acid stone in the left ureter. Diagnosed with Calculus of Ureter.
65. A 62-year-old female with a history of recurrent urinary tract infections complains of fever, chills, and right flank pain. CT scan reveals a 2cm renal abscess. Diagnosed with Acute Pyelonephritis with abscess.
66. A 35-year-old male presents with nocturia, hesitancy, and weak urinary stream. Digital rectal exam reveals an enlarged prostate. Diagnosed with Benign Prostatic Hyperplasia.

67. A 28-year-old female with a history of diabetes presents with dysuria, frequency, and suprapubic pain. Urine culture reveals Candida albicans. Diagnosed with Candidal Cystitis.

68. A 55-year-old male with a history of nephrolithiasis presents with severe right flank pain radiating to the groin. CT scan shows a 4mm obstructing calcium oxalate stone in the right ureter. Diagnosed with Calculus of Ureter.

69. A 48-year-old female presents with gross hematuria and left flank pain. Cystoscopy reveals a large, sessile tumor in the bladder. Biopsy shows high-grade papillary urothelial carcinoma. Diagnosed A 48-year-old female presents with gross hematuria and left flank pain. Cystoscopy reveals a large, sessile tumor in the bladder. Biopsy shows high-grade papillary urothelial carcinoma. Diagnosed with Muscle Invasive Bladder Cancer.
70. A 60-year-old male with a history of smoking presents with gross hematuria and weight loss. CT urography reveals a filling defect in the right renal pelvis. Diagnosed with Transitional Cell Carcinoma of Renal Pelvis.
71. A 30-year-old female presents with dysuria, frequency, and hematuria. Urine culture reveals Escherichia coli. Diagnosed with Acute Cystitis.
72. A 42-year-old male with a history of gout presents with severe left flank pain and vomiting. CT scan shows a 5mm obstructing uric acid stone in the left kidney. Diagnosed with Calculus of Kidney.
73. A 58-year-old female with a history of diabetes complains of right flank pain and fever. CT scan reveals emphysematous pyelonephritis. Diagnosed with Acute Emphysematous Pyelonephritis.
74. A 45-year-old male with a history of recurrent urinary tract infections presents with fever, chills, and left flank pain. Urine culture shows Pseudomonas aeruginosa. Diagnosed with Acute Pyelonephritis.
75. A 35-year-old female presents with severe right flank pain radiating to the groin. CT scan reveals a 3mm obstructing calcium oxalate stone in the right ureter. Diagnosed with Calculus of Ureter.
76. A 62-year-old male with a history of hypertension complains of nocturia, hesitancy, and weak urinary stream. Ultrasound reveals bilateral hydronephrosis. Diagnosed with Obstructive Uropathy due to Benign Prostatic Hyperplasia.
77. A 28-year-old female presents with dysuria, frequency, and suprapubic pain. Urine culture reveals Staphylococcus saprophyticus. Diagnosed with Acute Cystitis.
78. A 50-year-old male with a history of smoking presents with gross hematuria and flank pain. Cystoscopy reveals a small, papillary tumor in the bladder. Biopsy shows low-grade papillary urothelial carcinoma. Diagnosed with Non-Muscle Invasive Bladder Cancer.
79. A 55-year-old female with a history of nephrolithiasis presents with severe left flank pain and vomiting. CT scan shows a 6mm obstructing struvite stone in the left kidney. Diagnosed with Calculus of Kidney.
80. A 55-year-old female with a history of recurrent urinary tract infections presents with fever, chills, and left flank pain. Urine culture shows Pseudomonas aeruginosa. Diagnosed with Acute Pyelonephritis.

81. A 30-year-old male presents with severe right flank pain radiating to the groin. CT scan reveals a 3mm obstructing calcium oxalate stone in the right ureter. Diagnosed with Calculus of Ureter.
82. A 62-year-old male with a history of smoking presents with gross hematuria and flank pain. Cystoscopy reveals a large, sessile tumor in the bladder. Biopsy shows high-grade papillary urothelial carcinoma. Diagnosed with Muscle Invasive Bladder Cancer.
83. A 48-year-old female with a history of nephrolithiasis presents with severe left flank pain and vomiting. CT scan shows a 5mm obstructing struvite stone in the left kidney. Diagnosed with Calculus of Kidney.
84. A 35-year-old male presents with dysuria, frequency, and suprapubic pain. Urine culture reveals Staphylococcus saprophyticus. Diagnosed with Acute Cystitis.
85. A 60-year-old female with a history of diabetes complains of right flank pain and fever. CT scan reveals emphysematous pyelonephritis. Diagnosed with Acute Emphysematous Pyelonephritis.
86. A 28-year-old female with a history of recurrent urinary tract infections presents with fever, chills, and right flank pain. Urine culture shows Klebsiella pneumoniae. Diagnosed with Acute Pyelonephritis.
87. A 50-year-old male presents with severe left flank pain and hematuria. CT scan reveals a 4mm obstructing uric acid stone in the left ureter. Diagnosed with Calculus of Ureter.
88. A 55-year-old male with a history of hypertension complains of nocturia, hesitancy, and weak urinary stream. Ultrasound reveals bilateral hydronephrosis. Diagnosed with Obstructive Uropathy due to Benign Prostatic Hyperplasia.
89. A 42-year-old female presents with dysuria, frequency, and hematuria. Cystoscopy reveals a small, papillary tumor in the bladder. Biopsy shows low-grade papillary urothelial carcinoma. Diagnosed with Non-Muscle Invasive Bladder Cancer.
90. A 65-year-old male with a history of diabetes presents with fever, chills, and left flank pain. Urine culture shows Escherichia coli. Diagnosed with Acute Pyelonephritis.
91. A 30-year-old female with a history of nephrolithiasis presents with severe right flank pain and vomiting. CT scan reveals a 6mm obstructing calcium phosphate stone in the right kidney. Diagnosed with Calculus of Kidney.
92. A 58-year-old male with a history of smoking presents with gross hematuria and weight loss. CT urography reveals a large, irregular tumor in the left renal pelvis. Diagnosed with Transitional Cell Carcinoma of Renal Pelvis.
93. A 45-year-old female presents with dysuria, frequency, and suprapubic pain. Urine culture reveals Proteus mirabilis. Diagnosed with Acute Cystitis.
94. A 50-year-old male with a history of gout presents with severe left flank pain and hematuria. CT scan shows a 3mm obstructing uric acid stone in the left ureter. Diagnosed with Calculus of Ureter.
95. A 62-year-old female with a history of recurrent urinary tract infections complains of fever, chills, and right flank pain. CT scan reveals a 2cm renal abscess. Diagnosed with Acute Pyelonephritis with abscess.
96. A 35-year-old male presents with nocturia, hesitancy, and weak urinary stream. Digital rectal exam reveals an enlarged prostate. Diagnosed with Benign Prostatic Hyperplasia.

97. A 48-year-old female presents with gross hematuria and left flank pain. Cystoscopy reveals a large, sessile tumor in the bladder. Biopsy shows high-grade papillary urothelial carcinoma. Diagnosed with Muscle Invasive Bladder Cancer.
98. A 60-year-old male with a history of smoking presents with gross hematuria and weight loss. CT urography reveals a filling defect in the right renal pelvis. Diagnosed with Transitional Cell Carcinoma of Renal Pelvis.
99. A 45-year-old male with a history of recurrent urinary tract infections presents with fever, chills, and left flank pain. Urine culture shows Pseudomonas aeruginosa. Diagnosed with Acute Pyelonephritis.
100. A 55-year-old female with a history of nephrolithiasis presents with severe left flank pain and vomiting. CT scan shows a 6mm obstructing struvite stone in the left kidney. Diagnosed with Calculus of Kidney.

Key answers:

1. Q61.3
2. N17.9
3. M32.14
4. N04.9
5. E72.04
6. Q61.2
7. D59.3
8. E70.29
9. Q87.81
10. N18.4
11. E26.81
12. N20.0
13. M32.14
14. N10
15. E11.21
16. N04.1

17. N18.6
18. C64
19. M32.14
20. C65
21. N13.8
22. N20.1
23. D30.0
24. N40.1
25. N30.0
26. C67.9
27. C67.9
28. C65
29. D30.0
30. N40.0
31. B37.41
32. N13.8
33. C67.9
34. N20.1
35. N10
36. N40.0
37. B37.41
38. N20.1
39. C67.9
40. C65
41. N30.0

42. N20.0
43. N10
44. N10
45. N20.1
46. N13.8
47. N30.0
48. C67.9
49. N20.0
50. N10
51. N20.1
52. C67.9
53. N20.0
54. N30.0
55. N10
56. N10
57. N20.1
58. N13.8
59. C67.9
60. N10
61. N20.0
62. C65
63. N30.0
64. N20.1
65. N10
66. N40.0

67. B37.41
68. N20.1
69. C67.9
70. C65
71. N30.0
72. N20.0
73. N10
74. N10
75. N20.1
76. N13.8
77. N30.0
78. C67.9
79. N20.0
80. N10
81. N20.1
82. C67.9
83. N20.0
84. N30.0
85. N10
86. N10
87. N20.1
88. N13.8
89. C67.9
90. N10
91. N20.0

92. C65
93. N30.0
94. N20.1
95. N10
96. N40.0
97. C67.9
98. C65
99. N10
100. N20.0
